# Occurrences of Neuroptera and Raphidioptera in some regions in European Russia

**DOI:** 10.3897/BDJ.12.e135019

**Published:** 2024-10-18

**Authors:** Alexander Ruchin, Vladimir Makarkin, Mikhail Esin, Leonid Egorov, Oleg Artaev, Evgeniy Lobachev, Sergey Lukiyanov, Vasilii Anikin, Anatoliy Khapugin, Gennadiy Semishin

**Affiliations:** 1 Joint Directorate of the Mordovia State Nature Reserve and National Park "Smolny", Saransk, Russia Joint Directorate of the Mordovia State Nature Reserve and National Park "Smolny" Saransk Russia; 2 Federal Scientific Center of the East Asia Terrestrial Biodiversity, Far East Branch of the Russian Academy of Sciences, Vladivostok, Russia Federal Scientific Center of the East Asia Terrestrial Biodiversity, Far East Branch of the Russian Academy of Sciences Vladivostok Russia; 3 The State Nature Reserve "Prisursky", Cheboksary, Russia The State Nature Reserve "Prisursky" Cheboksary Russia; 4 Papanin Institute for Biology of Inland Waters Russian Academy of Sciences, Borok, Russia Papanin Institute for Biology of Inland Waters Russian Academy of Sciences Borok Russia; 5 National Research Mordovia State University, Saransk, Russia National Research Mordovia State University Saransk Russia; 6 Chernyshevsky Saratov State University, Saratov, Russia Chernyshevsky Saratov State University Saratov Russia; 7 Tyumen State University, Tyumen, Russia Tyumen State University Tyumen Russia

**Keywords:** dataset, observations, biodiversity, data paper

## Abstract

**Background:**

The document presents an extensive set of data on the occurrence of Neuroptera and Raphidioptera in some regions of European Russia. The results of our own research, as well as scientific collections, have been processed. The data were collected in 17 regions. In our own research, we used different ways to obtain information, which allowed us to collect extensive material for the dataset. This dataset provides valuable information about the biodiversity of Neuroptera and Raphidioptera, the abundance of each taxon collected and the time of taxon collections.

**New information:**

Our dataset contains up-to-date information on the occurrence of Neuroptera and Raphidioptera in the Volga River and Don River Basins located in the Russian Plain of European Russia (17 regions of European Russia). The dataset consists of 4,826 occurrence records. All of them are georeferenced (17,373 individuals were studied). A total of 83 species of Neuroptera (8 families, 36 genera) and four species of Raphidioptera (2 families, 4 genera) were recorded within the investigated area.

## Introduction

At present, the study and conservation of biological diversity are urgent global problems. Many species are on the verge of extinction, which is caused, amongst other factors, by anthropogenic activities that change the habitats of plants and animals. There are many causes that make habitats for living organisms change and disappear, for example, fires (Khosravi et al. 2022, Tiberio et al. 2022, Vilkova et al. 2023) and other natural catastrophic events (Vasilyev et al. 2023), environmental pollution (Sanders et al. 2023), impacts of biological invasions (Blackburn et al. 2019, Sanders et al. 2023) and direct human impacts (Lemoine et al. 2023, Mancini et al. 2023). Biodiversity conservation issues play an important role in protected area conservation programmes and development plans (e.g. Oliveira et al. (2022), Coutinho et al. (2023), Pham et al. (2023)). However, prior to conservation planning, biodiversity inventories are essential. This is particularly important for poorly-studied groups of plants and animals, such as Acari (Khaustov 2020), some insects (Esin et al. 2023) and other organisms, since many of these hidden, yet unknown, species may have become extinct before their discovery (Lees and Pimm 2015).

Neuroptera (lacewings), Megaloptera (dobsonflies, alderflies) and Raphidioptera (snakeflies) constitute the super-order Neuropterida, which is considered a sister of the Coleoptera and Strepsiptera (Plant 2007, Misof et al. 2014, Winterton et al. 2018). This super-order is a small group comprising about 6,500 described species (Aspöck 2002, Bowles 2015, Oswald 2023). The order Neuroptera includes about 6,000 species and the order Raphidioptera – no less than 240 species (Aspöck et al. 2015, Oswald and Machado 2018). Some representatives of these small orders have a certain importance in ecosystems as predators, regulating the populations of many pests (Solomon et al. 2000, Plant 2007, Silva et al. 2007). The study of these groups in the Middle Volga Region has been carried out since the 19^th^ century. However, the authors of this work have studied them most intensively since 2008, using a variety of methods (Makarkin and Ruchin 2014, Ruchin and Makarkin 2017, Makarkin and Egorov 2020, Makarkin and Ruchin 2020a, Makarkin and Ruchin 2020b, Makarkin and Ruchin 2021a, Makarkin and Ruchin 2021b, Makarkin and Ruchin 2021c).

This paper is aimed at describing a modern dataset on the occurrence of Neuroptera and Raphidioptera in the centre of European Russia, which has been recently published in GBIF as the Darwin Core Archive (Ruchin et al. 2023). This paper serves as a "data paper" (Penev et al. 2017).

## General description

### Additional information

All Neuroptera and Raphidioptera individuals were identified at the species level. The taxonomic diversity of the study area is represented by 87 species belonging to 10 families of two orders. Given the long-term nature of our research, this almost complete list of species (except the family Coniopterygidae) forms natural self-reproducing populations, although records of a few species new for the region occur regularly.

Some common species are distributed nearly continuously in this region, both in forests and open habitats (e.g. *Chrysoperlacarnea* (Stephens, 1836), *Hemerobiushumulinus* Linnaeus, 1758 and *Micromusangulatus* (Stephens, 1836)). Similarly, *Chrysopaperla* (Linnaeus, 1758) occurs almost everywhere, except steppes. Many species are more or less common over the region, but occur mainly on deciduous or conifer trees in the forests (e.g. *Ninetaflava* (Scopoli, 1763), *N.vittata* (Wesmael, 1841), *Apertochrysaflavifrons* (Brauer, 1851), *Cunctochrysaalbolineata* (Killington, 1935) and *C.cosmia* (Navás, 1918)). Other species prefer open habitats (e.g. *Psectradiptera* (Burmeister, 1839), *Chrysopaphyllochroma* Wesmael, 1841, *Ch.abbreviata* Curtis, 1834, *Ch.commata* Кis & Újhelyi, 1965, *Ch.dasyptera* McLachlan, 1872 and *Ch.walkeri* McLachlan, 1893).

In general, it is difficult to estimate the real abundance of lacewings in natural biocoenoses, as it largely depends on the collecting methods. For example, crown bait traps set on various deciduous trees and pines attract almost exclusively those species of lacewings that feed at the imaginal stage on pollen and nectar of flowers (phytophages) and honeydews (glycophages) (Makarkin and Ruchin 2019). Amongst them, three species clearly dominate (i.e. *Chrysopidiaciliata* (Wesmael, 1841), *Ninetaalpicola* (Kuwayama, 1956) and *Apertochrysaprasina* (Burmeister, 1839)), which constitute the vast majority of specimens of all lacewings collected by these traps. They probably occur everywhere in the forests of this region, but their relative abundance varies. *A.prasina* prefers more dry forests and it is very common in, for example, the Saratov, Ul’yanovsk and Astrakhan Oblasts, while *Ch.ciliata* and *N.alpicola* prefer more humid forests and they are rare in the south of the region (e.g. Samara and Saratov Oblasts; not recorded yet from the Astrakhan Oblast).

The known area of *Nothochrysafulviceps* (Stephens, 1836) in Russia is restricted only to the studied region (Makarkin and Ruchin 2023: fig. 2).

The population of *Chrysopaviridinervis* Jakowleff, 1869 in the Khvalynsk Forest (Saratov Oblast) is isolated. The nearest known localities of the species are the Donetsk Region and the North Caucasus.

The northern limit of the distribution of many southern species lies within this region. These are almost all species of Myrmeleontidae (except *Myrmeleonbore* (Tjeder, 1941) and *M.formicarius* Linnaeus, 1767), Ascalaphidae, Mantispidae (*Mantispaaphavexelte* U. Aspöck & H. Aspöck, 1994, *Mantispillaperla* (Pallas, 1772)) and a few Hemerobiidae (e.g. *Micromuslanosus* (Zelený, 1962)). Most of these occur in open habitats (steppe, forest-steppe, dry meadows), except for the Osmylidae and Hemerobiidae.

The occurrence in this region of three species (i.e. *Chrysoperlamutata* (McLachlan, 1898), *Wesmaeliusnavasi* (Andréu, 1911) and *W.vaillanti* (Navás, 1927)) is particularly noteworthy. These findings are northernmost in Europe.

To date, the eastern limit of the distribution for the following species lies within the region: *Hemerobiuslutescens* Fabricius, 1793, *H.micans*, *M.hirtus* (Linnaeus, 1761), *M.lanosus* (Zelený, 1962), *Sympherobiuselegans* and *S.pygmaeus*.

The occurrence of *Chrysopaviridana* and *Ch.nigricostata* in the region needs confirmation.

## Sampling methods

### Sampling description

Each observation contained fundamental information, such as location (coordinates), date, name of observer and name of identifier. A large part of the coordinates was determined directly on site using a GPS device. The margin of error in the measurement of coordinates is 50 m. When using references and data before 2007, the coordinates were indicated, which were processed on the Google map with reference to the locality indicated by the authors of the publications. At the same time, the accuracy of such data was up to 500 m. The accuracy of determining coordinates is up to the fourth digit. In all cases, the WGS 84 coordinate system is used.

To collect the material, we used fermental traps (Ruchin et al. 2020), as well as traps proposed by Jalas (1960), which were placed in the crowns of various trees at heights ranging from 1.5 to 12 m from the ground level. Fermented beer, sugar and honey served as bait (Ruchin et al. 2020). Yellow-coloured Mericke’s plates, hand collection with entomological nets and light trapping were also used.

## Geographic coverage

### Description

**Figure 1.**
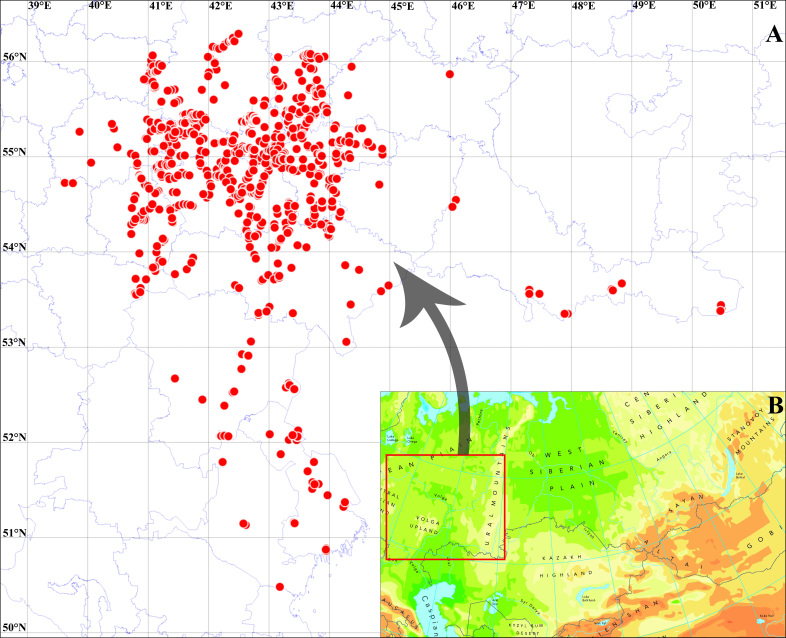
Collecting sites in the Volga River Basin and Don River Basin.

The dataset contains information on occurrences of Neuroptera and Raphidioptera taxa in 17 Russian regions, namely Chuvash Republic, Republic of Kalmykia, Republic of Mordovia, Mari El Republic, Republic of Tatarstan, Astrakhan Oblast, Nizhny Novgorod Oblast, Penza Oblast, Orenburg Oblast, Ryazan Oblast, Samara Oblast, Saratov Oblast, Tambov Oblast, Ulyanovsk Oblast, Vladimir Oblast, Volgograd Oblast and Voronezh Oblast (Fig. 1).

All collecting sites are located on the East European Plain. The main number of studied regions is situated on the Volga River Upland and Oka-Don Lowland, as well as Kalach Upland, Ergeni Upland, Don High Plain, Vyatsky Uval Upland, Mari Lowland, Peri-Caspian Lowland and Khoper-Buzuluk Plain. The north-to-south extent of the study area is more than 1300 km. In this regard, a number of climatic zones (i.e. taiga, zones of mixed forests, forest-steppe and steppe) are located within this area.

In the study area, the climate is moderate continental, with clearly distinct seasons (Milkov 1953, Egorov et al. 2022). The climate continentality increases in the northwest to southeast direction. In the northern part of the study area, the relative air humidity is higher, the summer is drier and hotter, while the winter is colder compared to the southern part.

In the study area, the watersheds of some large rivers of European Russia pass. The study area lies within the Black Sea Basin (watershed of the Don River) and the Caspian Sea Basin (watershed of the Volga River). In the study area, all rivers are of a typically low-lying type and belong to the Eastern European type. The relief is characterised by vast plain areas with altitudes of 130–250 m a.s.l. (up to 300 m a.s.l.). They alternate with wide valleys of large rivers, elongated in the meridional direction (e.g. Sura River, Moksha River, Don River, Voronezh River, Bityug River, Khoper River, Medveditsa River, Ilovlya River, Sviyaga River, Tsna River). In the northern part of the study area, swampy drainage lowlands are common.

### Coordinates

44°58'52"N and 56°36'21"N Latitude; 39°14'39"E and 60°58'28"E Longitude.

## Taxonomic coverage

### Description

There were 4,826 occurrence records published on Neuroptera and Raphidioptera from 17 regions of Russia: Chuvash Republic, Republic of Kalmykia, Republic of Mordovia, Republic of Tatarstan, Mari El Republic, Astrakhan Oblast, Nizhny Novgorod Oblast, Penza Oblast, Orenburg Oblast, Ryazan Oblast, Samara Oblast, Saratov Oblast, Tambov Oblast, Ulyanovsk Oblast, Vladimir Oblast, Volgograd Oblast and Voronezh Oblast. The authors of this dataset collected the records from 1987 to 2022. In addition, reliable data are presented from verified references that contain information on dates and localities of finds. We present a dataset that includes data on 83 species of Neuroptera and four species of Raphidioptera from natural and anthropogenic ecosystems. This is the first geographically referenced dataset on the occurrences of these insects for the European part of Russia. The classification of Neuropterida follows Oswald and Machado (2018); the nomenclature follows Oswald (2023).

Table 1 shows the taxonomic composition of the dataset, the number of observations and individuals and the number of regions where the species has been recorded.

### Taxa included

**Table:** 

Rank	Scientific Name	
phylum	Arthropoda	
class	Insecta	
order	Neuroptera	
family	Ascalaphidae	
family	Coniopterygidae	
family	Chrysopidae	
family	Hemerobiidae	
family	Mantispidae	
family	Myrmeleontidae	
family	Osmylidae	
family	Sisyridae	
order	Raphidioptera	
family	Inocelliidae	
family	Raphidiidae	

## Temporal coverage

**Formation period:** 1905, 1907, 1909, 1914, 1928, 1961, 1965, 1968, 1973, 1974, 1978, 1985-2022.

### Notes

**Figure 2.**
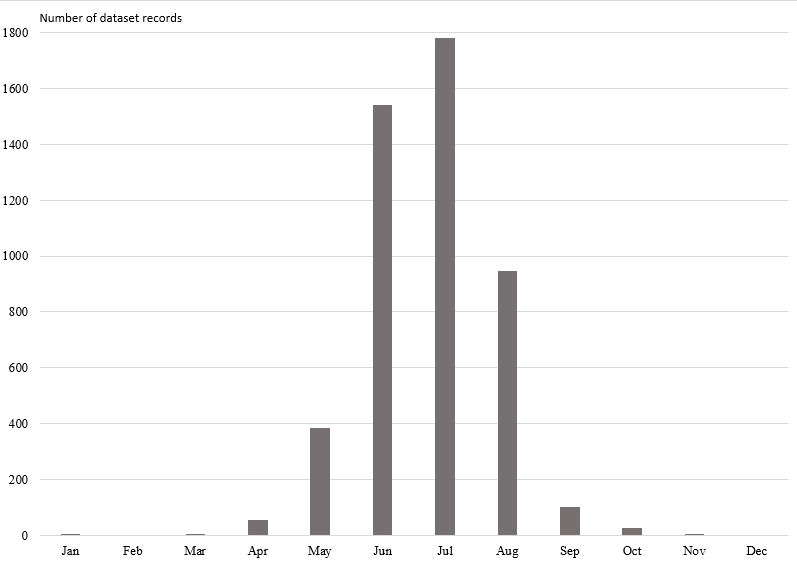
The number of occurrences of Neuroptera and Raphidioptera per month in the dataset.

The total number of occurrences in the dataset per month ranges from 0 in February and December to 1780 in July (Fig. 2). One occurrence in the dataset was made in March and November, two others were made in January. During these months, occurrences of wintering individuals were made in apartments and houses.

**Figure 3.**
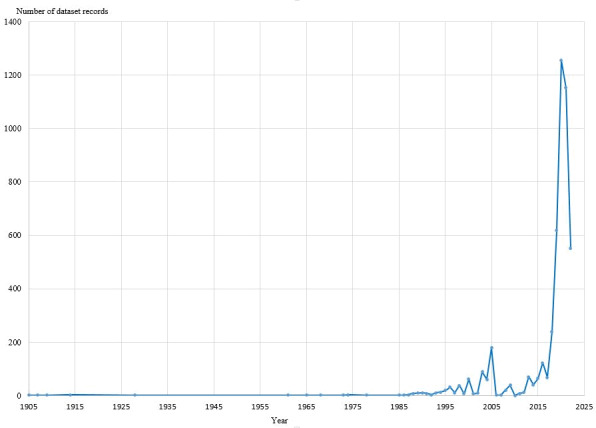
Total number of dataset occurrences of Neuroptera and Raphidioptera arranged for study years.

In the dataset, the main number of occurrences (3,577, 73.9%) was made in 2019–2022 (Fig. 3). During this period, a wide variety of research methods were used and the number of users increased considerably.

## Usage licence

### Usage licence

Other

### IP rights notes

CC-BY 4.0

## Data resources

### Data package title

Neuroptera and Raphidioptera in some regions of European part of Russia

### Resource link

https://doi.org/10.15468/f5ahps

### Alternative identifiers

https://www.gbif.org/dataset/b6f5b177-371b-46f2-a2db-3fbcd2aea828

### Number of data sets

1

### Data set 1.

#### Data set name

Neuroptera and Raphidioptera in some regions of European part of Russia

#### Data format

Darwin Core

#### Character set

UTF-8

#### Download URL

http://gbif.ru:8080/ipt/archive.do?r=neuroptera_raphidioptera_eururorussia

**Data set 1.** 

Column label	Column description
occurrenceID	An identifier for the Occurrence (as opposed to a particular digital record of the occurrence).
basisOfRecord	The specific nature of the data record: HumanObservation.
scientificName	The full scientific name including the genus name and the lowest level oftaxonomic rank with the authority.
kingdom	The full scientific name of the kingdom in which the taxon is classified.
phylum	The full scientific name of the phylum or division in which the taxon is classified.
class	The full scientific name of the class in which the taxon is classified.
decimalLatitude	The geographic latitude of location in decimal degrees.
decimalLongitude	The geographic longitude of location in decimal degrees.
coordinateUncertaintyInMetres	The horizontal distance (in metres) from the given decimalLatitude and decimal-Longitude describing the smallest circle containing the whole of the Location.
geodeticDatum	The ellipsoid, geodetic datum or spatial reference system (SRS) upon which the geographic coordinates given in decimalLatitude and decimalLongitude are based.
country	The name of the country in which the Location occurs. Here - Russia.
countryCode	The standard code for the country in which the Location occurs. Here - RU.
stateProvince	The name of the administrative region within country in which the Location occurs.
individualCount	The number of individuals represented present at the time of the Occurrence.
eventDate	The date when material from the trap was collected or the range of dates during which the trap collected material.
year	The integer year of the month on which the Event occurred.
month	The integer month on which the Event occurred.
day	The integer day of the month on which the Event occurred.
locality	The original textual description of the place.
georeferenceSources	A maps service used to georeference the location.
recordedBy	A person, group or organisation responsible for recording the original Occurrence.
identifiedBy	A list of names of people, who assigned the Taxon to the subject.
taxonRank	The taxonomic rank of the most specific name in the dwc:scientificName.
associatedReferences	A list of identifiers (publication, bibliographic reference, global unique identifier, URI) of literature associated with the dwc:Occurrence.
occurrencesRemarks	Comments or notes about the dwc:Occurrence.
habitat	A category or description of the habitat in which the dwc:Event occurred.
samplingProtocol	The methods or protocols used during a dwc:Event.

## Additional information

### Observers

Seventy-eight observers contributed to the dataset, of which 42 (53.8%) contributed to more than one dataset record. Additionally, 18 (23.1%) observers contributed to more than ten occurrences in the dataset (Table 2). When using one or two research methods, data from one user may not have a significant contribution to the overall fauna of a region. However, such data from a single user are useful for understanding the general distribution of species.

## Figures and Tables

**Table 1.** Taxonomic composition of the dataset, number of observations and individuals.

Taxa	Number of observations	Number of specimens	Number of regions where the species has been recorded
**NEUROPTERA**			
**Coniopterygidae**			
*Aleuropteryxloewii* Klapálek, 1894	1	1	1
*Coniopteryxpygmaea* Enderlein, 1906	6	43	1
*Coniopteryxtineiformis* Curtis, 1834	1	1	1
*Conwentziapineticola* Enderlein, 1905	2	2	2
*Parasemidalisfuscipennis* (Reuter, 1894)	3	3	3
*Semidalisaleyrodiformis* (Stephens, 1836)	9	16	5
**Sisyridae**			
*Sisyranigra* (Retzius, 1783)	35	230	5
*Sisyraterminalis* Curtis, 1854	5	7	2
**Ascalaphidae**			
*Libelloidesmacaronius* (Scopoli, 1763)	21	48	4
**Osmylidae**			
*Osmylusfulvicephalus* (Scopoli, 1763)	5	12	1
**Hemerobiidae**			
*Drepanepteryxalgida* (Erichson, 1851)	1	1	1
*Drepanepteryxphalaenoides* (Linnaeus, 1758)	12	12	3
*Hemerobiusatrifrons* McLachlan, 1868	1	1	1
*Hemerobiushumulinus* Linnaeus, 1758	59	72	11
*Hemerobiuslutescens* Fabricius, 1793	3	3	2
*Hemerobiusmarginatus* Stephens, 1836	11	13	4
*Hemerobiusmicans* Olivier, 1793	7	10	3
*Hemerobiusnitidulus* Fabricius, 1777	25	55	6
*Hemerobiuspini* Leach, 1815	1	1	1
*Hemerobiussimulans* Walker, 1853	5	7	2
*Hemerobiusstigma* Stephens, 1836	9	9	3
*Hemerobiusstriatus* Nakahara, 1915	4	7	1
*Megalomushirtus* (Linnaeus, 1761)	19	23	3
*Micromusangulatus* (Stephens, 1836)	94	131	10
*Micromuslanosus* (Zelený, 1962)	1	1	1
*Micromuspaganus* (Linnaeus, 1767)	4	4	3
*Micromusvariegatus* (Fabricius, 1793)	33	51	5
*Psectradiptera* (Burmeister, 1839)	11	16	4
*Sympherobiuselegans* (Stephens, 1836)	2	2	2
*Sympherobiusfuscescens* (Wallengren, 1863)	1	1	1
*Sympherobiuspygmaeus* (Rambur, 1842)	4	6	4
*Wesmaeliusconcinnus* (Stephens, 1836)	9	10	3
*Wesmaeliusmortoni* (McLachlan, 1899)	2	2	1
*Wesmaeliusnavasi* (Andréu, 1911)	1	1	1
*Wesmaeliusnervosus* (Fabricius, 1793)	8	10	2
*Wesmaeliusvaillanti* (Navás, 1927)	1	1	1
**Chrysopidae**			
*Apertochrysaflavifrons* (Brauer, 1851)	178	418	10
*Apertochrysaprasina* (Burmeister, 1839)	836	5371	16
*Apertochrysaventralis* (Curtis, 1834)	149	223	8
*Chrysopaabbreviata* Curtis, 1834	76	135	9
*Chrysopacommata* Кis & Újhelyi, 1965	80	272	10
*Chrysopadasyptera* McLachlan, 1872	18	27	2
*Chrysopadorsalis* Burmeister, 1839	18	29	6
*Chrysopadubitans* McLachlan, 1887	5	5	4
*Chrysopaformosa* Brauer, 1851	33	61	8
*Chrysopagibeauxi* (Leraut, 1989)	158	321	10
*Chrysopahummeli* Tjeder, 1936	21	38	4
*Chrysopanigricostata* Brauer, 1851	1	1	1
*Chrysopapallens* (Rambur, 1838)	21	28	9
*Chrysopaperla* (Linnaeus, 1758)	394	1300	11
*Chrysopaphyllochroma* Wesmael, 1841	58	124	9
*Chrysopaviridana* Schneider, 1845	1	3	1
*Chrysopaviridinervis* Jakowleff, 1869	3	3	1
*Chrysopawalkeri* McLachlan, 1893	105	180	8
*Chrysoperlacarnea* (Stephens, 1836)	493	1863	16
*Chrysoperlamutata* (McLachlan, 1898)	1	1	1
*Chrysopidiaciliata* (Wesmael, 1841)	469	2110	10
*Cunctochrysaalbolineata* (Killington, 1935)	36	46	7
*Cunctochrysacosmia* (Navás, 1918)	29	48	8
*Ninetaalpicola* (Kuwayama, 1956)	493	2039	10
*Ninetaflava* (Scopoli, 1763)	135	298	12
*Ninetavittata* (Wesmael, 1841)	94	146	8
*Nothochrysafulviceps* (Stephens, 1836)	52	124	8
**Mantispidae**			
*Mantispaaphavexelte* U. Aspöck & H. Aspöck, 1994 (=*Mantispalobata* Navás, 1912 sensu Zakharenko, 1987) *Mantispastyriaca* (Poda, 1761)	7 22	52 57	4 7
*Mantispillaperla* (Pallas, 1772)	3	3	3
**Myrmeleontidae**			
*Acanthaclisisoccitanica* (Villers, 1789)	14	37	4
*Creoleonplumbeus* (Olivier, 1811)	10	20	4
*Deutoleonlineatus* (Fabricius, 1798)	21	76	4
*Distoleontetragrammicus* (Fabricius, 1798)	32	214	6
*Euroleonnostras* (Geoffroy, 1785)	3	4	1
*Lopezusfedtschenkoi* (McLachlan, 1875)	2	2	1
*Macronemurusbilineatus* Brauer, 1868	3	4	2
*Megistopusflavicornis* (Rossi, 1790)	16	22	6
*Mesonemurusguentheri* Hölzel, 1970	2	7	1
*Myrmecaelurustrigrammus* (Pallas, 1771)	45	262	7
*Myrmecaelurusuralensis* (Hölzel, 1969)	6	8	2
*Myrmeleonbore* (Tjeder, 1941)	438	111	7
*Myrmeleonformicarius* Linnaeus, 1767	60	115	11
*Myrmeleonimmanis* Walker, 1853	19	62	4
*Myrmeleoninconspicuus* Rambur, 1842	25	55	4
*Neuroleonnemausiensis* (Borkhausen, 1791)	5	8	2
*Myrmecaeluruszigan* (H. Aspöck et al. 1980)	13	50	4
**RAPHIDIOPTERA**			
**Raphidiidae**			
*Dichrostigmaflavipes* (Stein, 1863)	85	118	8
*Raphidiaophiopsis* Linnaeus, 1758	6	29	3
*Xanthostigmaxanthostigma* (Schummel, 1832)	12	26	5
**Inocelliidae**			
*Inocelliacrassicornis* (Schummel, 1832)	4	4	4
**TOTAL**	4,826	17,373	

**Table 2.** Number of observations associated with a number of collectors in the dataset.

Number of Observations	Number of users
1	36
2–10	24
11–50	10
51–100	2
101–500	5
> 500	1
